# Plasmonic Nanopore Sensing to Probe the DNA Loading Status of Adeno-Associated Viruses

**DOI:** 10.3390/chemosensors13120418

**Published:** 2025-12-04

**Authors:** Scott Renkes, Steven J. Gray, Minjun Kim, George Alexandrakis

**Affiliations:** 1 Bioengineering Department, University of Texas at Arlington, Arlington, TX 76010, USA; 2 Department of Pediatrics, University of Texas Southwestern, Dallas, TX 75390, USA; 3 Department of Mechanical Engineering, Southern Methodist University, Dallas, TX 75205, USA

**Keywords:** plasmonic nanopore, adeno-associated virus, single-particle sensing, GFP, optical–electrical detection, AAVs, gene therapy quality control

## Abstract

Adeno-associated viruses (AAVs) are a leading vector for gene therapy, yet their clinical utility is limited by the lack of robust quality control methods to distinguish between empty (AAV_empty_), partially loaded (AAV_partial_), and fully DNA loaded (AAV_full_) capsids. Current analytical techniques provide partial insights but remain limited in sensitivity, throughput, or resolution. Here we present a multimodal plasmonic nanopore sensor that integrates optical trapping with electrical resistive-pulse sensing to characterize AAV9 capsids at the single-particle level in tens of μL sample volumes and fM range concentrations. As a model system, we employed AAV9 capsids not loaded with DNA, capsids loaded with a self-complementary 4.7 kbp DNA (AAV_scDNA_), and ones loaded with single-stranded 4.7 kbp DNA (AAV_ssDNA_). Ground-truth validation was performed with analytical ultracentrifugation (AUC). Nanosensor data were acquired concurrently for optical step changes (occurring at AAV trapping and un-trapping) both in transmittance and reflectance geometries, and electrical nanopore resistive pulse signatures, making for a total of five data dimensions. The acquired data was then filtered and clustered by Gaussian mixture models (GMMs), accompanied by spectral clustering stability analysis, to successfully separate between AAV species based on their DNA load status (AAV_empty_, AAV_partial_, AAV_full_) and DNA load type (AAV_scDNA_ versus AAV_ssDNA_). The motivation for quantifying the AAV_empty_ and AAV_partial_ population fractions is that they reduce treatment efficacy and increase immunogenicity. Likewise, the motivation to identify AAV_scDNA_ population fractions is that these have much higher transfection rates. Importantly, the results showed that the nanosensor could differentiate between AAV_scDNA_ and AAV_ssDNA_ despite their identical masses. In contrast, AUC could not differentiate between AAV_scDNA_ and AAV_ssDNA_. An equimolar mixture of AAV_scDNA_, AAV_ssDNA_ and AAV_empty_ was also measured with the sensor, and the results showed the expected population fractions, supporting the capacity of the method to differentiate AAV load status in heterogeneous solutions. In addition, less common optical and electrical signal signatures were identified in the acquired data, which were attributed to debris, rapid entry re-entry to the optical trap, or weak optical trap exits, representing critical artifacts to recognize for correct interpretation of the data. Together, these findings establish plasmonic nanopore sensing as a promising platform for quantifying AAV DNA loading status and genome type with the potential to extend ultra-sensitive single-particle characterization beyond the capabilities of existing methods.

## 1. Introduction

Gene therapy holds promise for treating over 10,000 monogenic disorders world-wide [1] and is now being explored for use in more complex polygenic disorders, broadening its therapeutic reach [2,3]. However, reliable vector quality control (QC) remains a major barrier to clinical translation [4]. Adeno-associated viruses (AAVs) have emerged as the leading delivery vehicle, with more than 250 ongoing human trials [5] and 7 FDA-approved treatments already in use, including Luxturna and Zolgensma, with dozens more in the regulatory pipeline [6]. A critical challenge in AAV manufacturing is the accurate determination of DNA loading status within capsids [7]. Key metrics—such as capsid and genome titers, and the population fractions of empty (AAV_empty_), partially filled with DNA (AAV_partial_), or fully loaded capsids (AAV_full_)—are difficult to assess with existing technologies [8]. Accurately determining capsid fill efficiency is essential for precise dosing, as under-filled vectors can reduce therapeutic potency while over-dosed preparations increase the risk of toxicity, immune activation, and other adverse events. In addition to distinguishing genome load, the ability to discriminate between genome forms such as single-stranded DNA (ssDNA) versus self-complementary DNA (scDNA) is crucial because ssDNA vectors are more immunogenic [9]. Furthermore, ssDNA vectors require second-strand synthesis before expression, whereas scDNA vectors bypass this rate-limiting step and enable faster, more efficient transgene expression [10].

Several analytical approaches are currently employed to characterize AAVs, each with distinct strengths and limitations. Analytical ultracentrifugation (AUC) is considered the most reliable method for distinguishing empty, partial, and full capsids, as well as detecting aggregates and free nucleic acids [11]. Quantitative polymerase chain reaction (qPCR) or digital droplet PCR (ddPCR) are used to quantify genome titer [12], and enzyme-linked immunosorbent assay (ELISA) is used for capsid titer [13]. Dynamic light scattering (DLS) can provide rapid and reproducible measurements of particle size distribution and aggregation [14,15], but it lacks the resolution required to distinguish subtle differences between particles of different DNA load but similar AAV size. Mass spectrometry (MS), including native and charge-detection approaches, enables sensitive analysis of capsid content and offers orthogonal validation of genome composition [16]. More recently, new technologies have entered the AAV QC field, including the Stunner system (Unchained Labs) that operates based on the thermo-diffusion properties of different nanoparticles [17], and mass photometry (Samux MP, Refeyn) that offers sensitive discrimination between AAV particles of different mass [18,19]. Despite these advances, attaining comprehensive, high-sensitivity, low sample volume QC of AAV particles in heterogeneous solutions remains a challenge. Variability in the ability to estimate the AAV_empty_, AAV_partial_, and AAV_full_ population fractions in biomanufactured AAV solutions, which are unavoidably heterogeneous, by current QC assays can result in overdosing, where empty capsids trigger immune responses, or underdosing, which limits therapeutic efficacy despite curative potential.

To improve discrimination capacity between AAV particles with different DNA load status (AAV_empty_, AAV_partial_, and AAV_full_) and DNA load cargo type (AAV_scDNA_ versus AAV_ssDNA_), we have developed a plasmonic nanopore sensor that integrates optical trapping with nanopore resistive-pulse sensing for single-nanoparticle characterization. The device consists of a solid-state nanopore embedded within a plasmonic nanostructure, enabling simultaneous optical and electrical readout of individual nanoparticles, proteins and protein complexes, as reported previously [20–22]. In this work, we report further development of this nanosensor technology for discriminating between AAV9 particles with different DNA load status and type, label-free, at single particle resolution. Specifically, by analyzing the concurrently acquired optical-electrical data with machine-learning, this sensor enabled sensitive discrimination between AAV_scDNA_ and AAV_ssDNA_, even though they are designed to have identical mass. This was accomplished by probing the effects of DNA conformation on capsid deformation. Differences in the conformation of the ssDNA versus scDNA genomes can influence the overall shape and rigidity of the AAV9 capsid. These conformational differences also shift the distribution of internal charges, which in turn modify the capsid’s effective dielectric properties and surface charge, producing distinct optical and electrical signatures. Furthermore, the nanosensor measurements could discriminate between AAV_empty_, AAV_partial_, and AAV_full_ in solutions containing only AAV_scDNA_ or AAV_ssDNA_, and in an equimolar mixture of AAV_empty_, AAV_scDNA_ and AAV_ssDNA_. Additionally, these measurements were performed at 50 μL sample volumes and fM AAV concentrations. These results support the promise of this plasmonic nanosensor technology as a new path forward towards more sensitive QC for gene therapy formulations.

## 2. Materials and Methods

### 2.1. Sensor Fabrication

Plasmonic nanopore sensors were fabricated from intrinsic 〈100〉 orientation silicon (Si) wafers, 4-inch diameter, 525 ± 25 μm thick (Nova Electronic Materials, Flower Mound, TX, USA). A 500 nm thermal oxide layer (SiO_2_) was grown using a Tytan series thermal oxidation furnace (Tystar Corporation, Garden Grove, CA, USA). Subsequently, a 60 nm silicon nitride (Si_x_N_y_) film was deposited by low-pressure chemical vapor deposition (LPCVD) in the Tytan LPCVD system (Tystar Corporation, Garden Grove, CA, USA). For the plasmonic metal layer, wafers were coated with a negative photoresist (NR7–1500PY, Futurrex, Franklin, NJ, USA) and patterned using a backside mask aligner (Model 806 Manual Front/Back Contact Mask Aligner Series 800, OAI, Milpitas, CA, USA). After development in RD-6 (Futurrex, Franklin, NJ, USA) and an oxygen plasma descum (Trion DRIE system, Trion Technology, Clearwater, FL, USA), a 5 nm Chromium (Cr) adhesion layer and 100 nm gold (Au) layer were deposited using an ATC Orion series electron-beam evaporation system (AJA International Inc., Hingham, MA, USA). Lift-off in sonication yielded patterned Au features. For backside patterning, wafers were coated with a positive photoresist (PR1–1000A; Futurrex, Franklin, NJ, USA) and again aligned with the OAI 806 mask aligner. After development in RD-6 and an oxygen descum (Trion DRIE system, Clearwater, FL, USA), the exposed Si_x_N_y_ and SiO_2_ layers were etched using a CF_4_ plasma (Trion DRIE system, Clearwater, FL, USA) to open access windows through to the silicon substrate.

The wafers were then loaded into an illuminated wafer holder (ART0042 IL-HAL, Advanced Micromachining Tools, Frankenthal, Germany) designed for 4-inch wafers and immersed in a 40% potassium hydroxide (KOH) bath at 80 °C for ~7 h to anisotropically etch through the intrinsic Si. Once optical transmission through the windows confirmed complete breakthrough, the etch was extended by an additional ~2 h to ensure removal of residual SiO_2_ and ensure the membrane was released. Wafers were rinsed and cleaned before being separated into individual chips. Cleaving was performed manually with a glass scribe, leveraging cleavage lines defined during the nitride etch. In some cases, these lines were further deepened by KOH etching to facilitate controlled fracture and clean chip separation.

**Figure 1.**
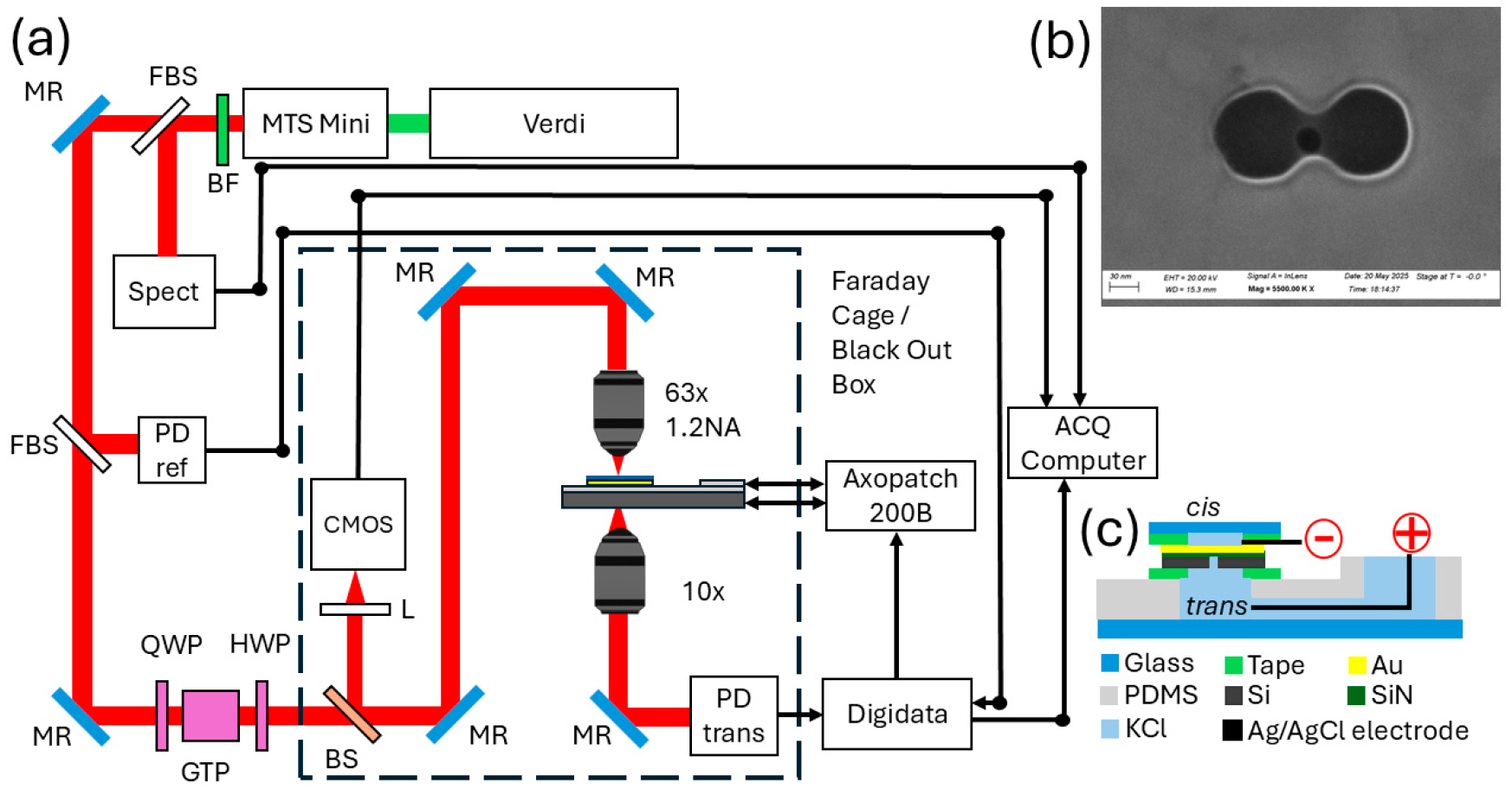
(**a**) Experimental setup. (**b**) Plasmonic nanopore at 5.5 M X magnification. (**c**) Flow cell assembly.

Final nanostructuring was performed at Oak Ridge National Laboratory using a focused ion beam helium ion (He-Ne) microscope (ORION NanoFab Helium Ion Microscope; Carl Zeiss SMT, Oberkochen, Germany). Double nanohole (DNH) antennas were milled using the neon beam, producing apertures ~100 nm in diameter with cusp spacing of ~42 nm. A single nanopore was then milled at the center of each DNH using the helium ion beam, targeting a diameter of ~30 nm (typical diameter achieved ~32 nm). The resulting structure is shown in Figure 1b.

The DNH geometry was selected to enable self-induced back action (SIBA) trapping for nanoparticles in the 5–50 nm size range, which includes the ~25 nm AAV9 capsid [23]. The SIBA effect occurs when a nanoparticle or molecule trapped near a plasmonic nanostructure modifies the local optical field in a way that dynamically enhances and stabilizes its own trapping potential through feedback between the particle’s position and the resonator’s optical response [24]. The gap in the DNH concentrates the electromagnetic field into a highly confined hotspot, enabling stable SIBA trapping with low optical power. The aperture diameter (~100 nm) and cusp spacing (~42 nm) were chosen based on prior work by Chen et al. [25], which demonstrated that this DNH geometry supports a plasmonic resonance near 820 nm. This matches the trapping laser wavelength used in our system and therefore maximizes field enhancement and trapping efficiency at the nanopore.

Photomasks used in both lithography steps were designed in-house and fabricated by the Nanotech Institute at the University of Texas at Dallas.

### 2.2. Optical-Electrical Plasmonic Nanopore Sensor Setup

The experimental platform consisted of a dual-mode optical and electrical detection system centered on a plasmonic nanopore (Figure 1). Optical trapping was achieved using an MTS Mini Ti:Sapphire laser operated in continuous-wave (CW) mode tuned at 820 nm with ~300 mW output power (KMLabs, Boulder, CO, USA). A 5 W Verdi pump laser at 532 nm provided excitation for the Ti:Sapphire cavity (Coherent, Saxonburg, PA, USA). Residual pump light was removed with an ET665lp barrier long-pass filter (Chroma Technology Corp., Bellows Falls, VT, USA) shown as BF in Figure 1a.

Along the optical path after the laser source, a 1^′′^ diameter BSF10-B fused silica beam sampler (FBS in Figure 1a) provided a ~1% diagnostic pick-off to a spectrometer (Ocean Optics, Orlando, FL, USA) shown as Spect in Figure 1a. A second fused silica sampler directed light to a PDA36A2 amplified photodiode (Thorlabs, Newton, NJ, USA), shown as PD ref in Figure 1a, for intensity tracking. Polarization was controlled using a WPQ05M-808 zero-order quarter-wave plate (optimized for 780–820 nm), a GTH10M Glan–Thompson polarizer (transmission 350 nm–2.3 μm), and a WPH05M-830 zero-order half-wave plate (optimized for 780–830 nm) (Thorlabs, Newton, NJ, USA) shown as QWP, GTP and HWP in Figure 1a, respectively. A beam splitter (BS) in Figure 1a transmitted the trapping beam to the sensing plane while routing back-reflected light to a CS135MUN CMOS camera (Thorlabs, Newton, NJ, USA) shown as CMOS in Figure 1a, for optical alignment.

The beam path was then raised with a periscope and focused onto the nanosensor using a C-Apochromat 63×/1.2 W Corr water-immersion apochromat objective (Carl Zeiss Microscopy GmbH, Jena, Germany). Alignment to the DNH sensor was guided by fiducial markers patterned during chip fabrication and finalized by adjusting the half-wave plate to maximize transmitted intensity. On the transmission side, light was collected with an RMS10X 10× dry objective, NA 0.25, WD 10.6 mm (Thorlabs, Newton, NJ, USA) and focused onto a second PDA36A2 photodiode (Thorlabs, Newton, NJ, USA) shown as PD trans in Figure 1a.

Ionic currents were measured using an Axopatch 200B patch-clamp amplifier and digitized with a Digidata 1500B interface (Molecular Devices, San Jose, CA, USA). During all trapping experiments, a +110 mV command voltage was applied across the nanopore.

### 2.3. Flow Cell Fabrication and Assembly

Flow cells were fabricated using polydimethylsiloxane (PDMS; Sylgard 184, Dow Corning, Midland, MI, USA). PDMS was cast in a custom-machined aluminum mold (Hubs) and allowed to cure overnight at 65 °C. After curing, the PDMS block was demolded, washed with isopropanol, and bonded to a glass slide via corona plasma treatment (BD-20; Electro-Technic Products, Inc., Chicago, IL, USA) for strong adhesion. The assembled flow cell was mounted into a custom 3D-printed caddy to interface with the microscope stage. Nanopore chips were affixed to the PDMS chamber using SecureSeal Adhesive Sheets (SAS-1L, 120 μm thickness; Grace Bio-Labs, Bend, OR, USA). Over the chip, a 170 μm glass coverslip was bonded via SecureSeal SAS-2L (240 μm thickness), ensuring the sensor region lies within the 270 μm working distance of the Zeiss 63× water-immersion objective. A schematic of the flow cell assembly is shown in Figure 1c. For the experiments, the *trans* chamber was filled with 300 mM KCl electrolyte. Before sealing the *cis* side with the coverslip, 50 μL of sample was applied directly onto the sensor surface also diluted in 300 mM KCl.

### 2.4. AAV Constructs and Sample Preparation

A non-therapeutic model of AAV9 capsids was loaded with different forms of the green fluorescent protein (GFP) gene as provided by the University of Texas Southwestern (UTSW) AAV Core. The GFP load serves as an effective analog genome for characterizing AAV loading efficiency and when used in cell culture, the resulting green glow from successful transfections can be used as an indicator of transfection efficiency. Single-stranded (AAV_ssDNA_) and self-complementary (AAV_scDNA_) vectors were produced separately, each packaging a GFP transgene expression cassette. The cassette fit within the 2.2 kb size limit of AAV_scDNA_ particles. To generate AAV_ssDNA_, an additional 2.2 kb of stuffer DNA was included. The two plasmid constructs used for AAV production differed only in one inverted terminal repeat (ITR, wild type for AAV_ssDNA_, mutated for AAV_scDNA_) and the presence of the stuffer sequence. Once packaged, both vectors contained a total ssDNA length of ~4.7 kb and so had a near-identical total mass, but different internal DNA load conformations. As a note, AAV9 particles have a diameter of ~25 nm and can be slightly asymmetrical in three dimensions due to their deformability, depending on capsid protein composition [26]. The DNA load status for all AAV9 solutions was validated against reference standards available at the UTSW AAV Core [8,27], namely AUC to separate empty from filled capsids and gel electrophoresis to verify DNA load integrity. The same AAV9 preparations and their detailed characterization by AUC has been reported on in prior work [28]. The variability of AAV sizes is attributed to the range of DNA load sizes from full, or even over-full, genomes to partial genomes, small fragments, or combinations thereof, as observed, for example, in gel electrophoresis smear patterns [29].

The samples were provided in 20 μL aliquots of 490 nM, 183 nM and 61 nM of AAV_empty_, AAV_scDNA_ and AAV_ssDNA_, respectively. All samples were diluted to 10 fM in 300 mM KCl in 100:1 dilution steps prior to measurement and pipetted in the 50 μL PDMS flow cell holding the sensor. For the experiments with heterogeneous solutions, equimolar mixtures were prepared by combining 100 μL of each single-species solution (10 fM), resulting in a final equimolar concentration of 3.33 fM per particle type (AAV_empty_, AAVscDNA, AAVssDNA).

### 2.5. Event Detection and Preprocessing

**Figure 2.**
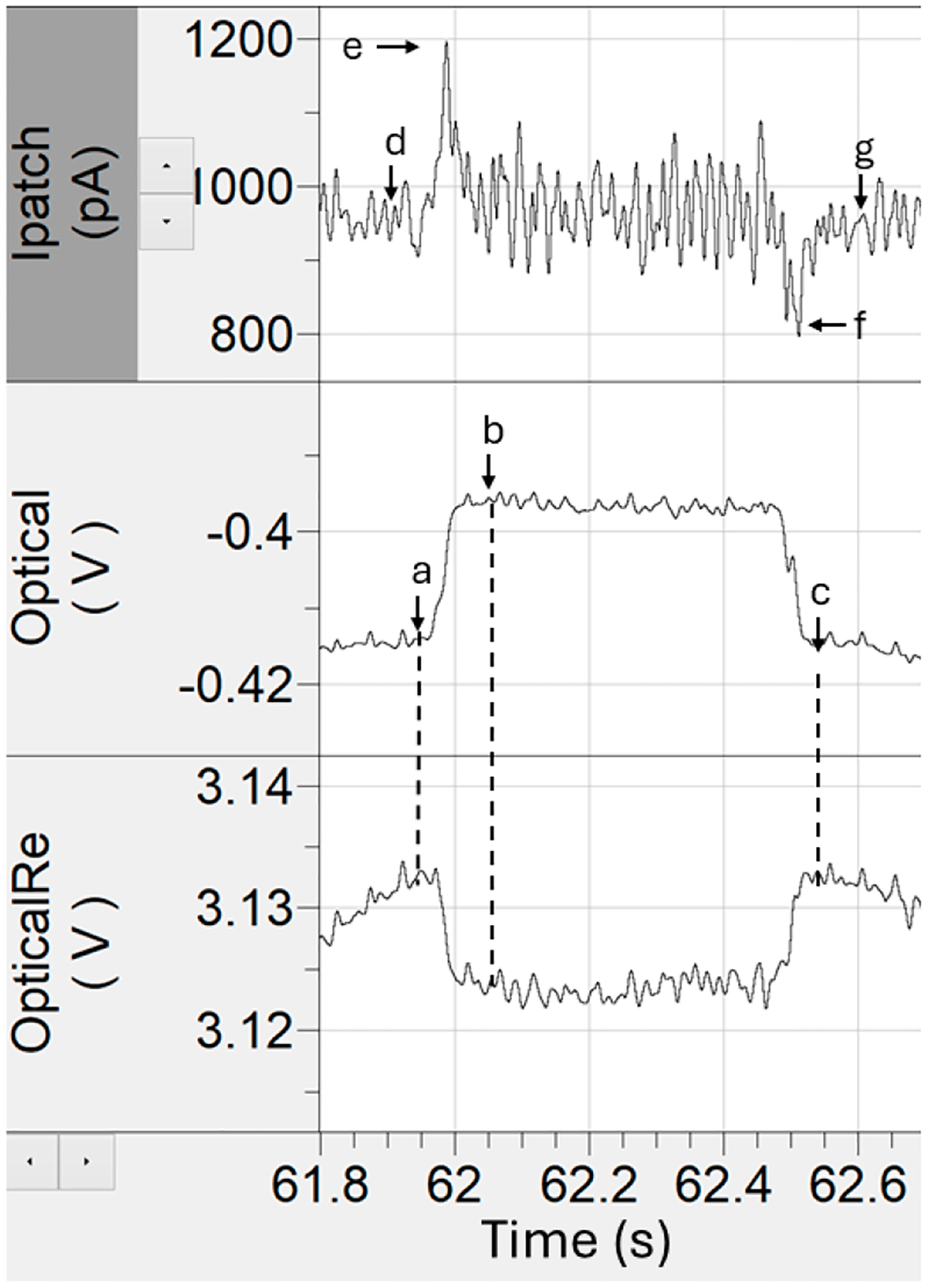
Data landmarks used for tagging optical and electrical time-series data features.

Raw optical and electrical time series were first filtered with a digital 8-pole Bessel filter with a 50 Hz cutoff frequency to reduce high-frequency noise before event tagging. Event identification was guided by the annotated schematic in Figure 2. Points a and b marked the pre- and post-event optical signal levels and were used to calculate the optical step change in the signal. The time interval between points a and c defined the trapping duration. Point d represented the baseline electrical current prior to optical trapping, while points e and f corresponded to the peaks of the electrical entry and exit spikes, respectively. Point g indicated the post-event electrical baseline. Together, these parameters captured both optical and electrical signatures of each trapping event. To minimize artifacts, events were screened using z-score trimming. The z-score for each event feature was calculated using Equation (1):

(1) $$z=\frac{x-\mu }{\sigma }$$

In this equation, *x* is the feature value, μ is the mean, and *σ* is the standard deviation of the full dataset. Events with |z| > 5 were discarded as outliers. This z-correction step effectively removed spurious events such as large aggregates, electrical noise spikes, and manual mis-tagging errors introduced during event annotation. We note that this method assumes a unimodal Gaussian distribution, whereas our event features were often multimodal. Consequently, trimming was applied relative to the overall mean and was more effective at excluding large deviations than at rejecting subtle outliers closer to zero amplitude.

### 2.6. Statistical Analysis and Clustering

Univariate event distributions were modeled with Gaussian mixture models (GMMs) to identify subpopulations [30,31]. For multidimensional analysis, event features were min–max normalized across five dimensions: Trapping duration, Transmission optical step change, Reflectance optical step change, Electrical entry spike amplitude, Electrical exit spike amplitude. Spectral clustering was then applied across the five normalized dimensions following established approaches [32,33]. To evaluate stability, clustering was repeated multiple times for each candidate cluster number *k*, and stability was quantified using the mean pairwise Normalized Mutual Information (NMI), defined in Equation (2). The minimum useful (separable) number of clusters was identified as the *k* that maximized the mean NMI (Equation (3)). This stability-based approach to model selection follows principles described by Volkovich and Avros [34]. The theoretical consistency of spectral clustering under sampling assumptions has also been demonstrated in the variational framework of García Trillos and Slepčev [35]. Stability curves $\bar{\text{NMI}}(k)$ (Equation (2)) were plotted as a function of the number of clusters *k* (Equation (3)) along with the computed stability uncertainty (±1 SD). Inflection points or plateauing behavior in $\bar{\text{NMI}}(k)$ with increasing *k* were interpreted as diminishing returns in increased stability at the cost of parsing data into a higher number of clusters.


(2) $$\text{NMI}(A,B)=\frac{I(A;B)}{\sqrt{H(A)\;H(B)}}$$

(3) $$k^{⋆}=\underset{\text{k}\in 𝒦}{\text{argmax}}\;\bar{\text{NMI}}(k)$$

To optimize the clustering, we incremented *k* stepwise, starting at the minimum useful cluster described above, and tracked how clusters evolved across iterations. Clusters that persisted with high membership overlap across successive *k* values were deemed stable and retained. Conversely, when increasing *k* produced new clusters that were roughly even splits of previously stable groups, daughter clusters with similar sizes and near-identical feature distributions, we treated this as evidence of over-clustering (arbitrary subdivision rather than discovery of new structure). In such cases, we stepped back to the last *k* at which all retained clusters remained stable and well separated.

For visualization, clusters were displayed both in the two most separable original features (transmission and reflectance step changes) and in the eigenspace projection defined by spectral clustering.

## 3. Results

### 3.1. Gaussian Mixture Model Analysis (GMM)

**Figure 3.**
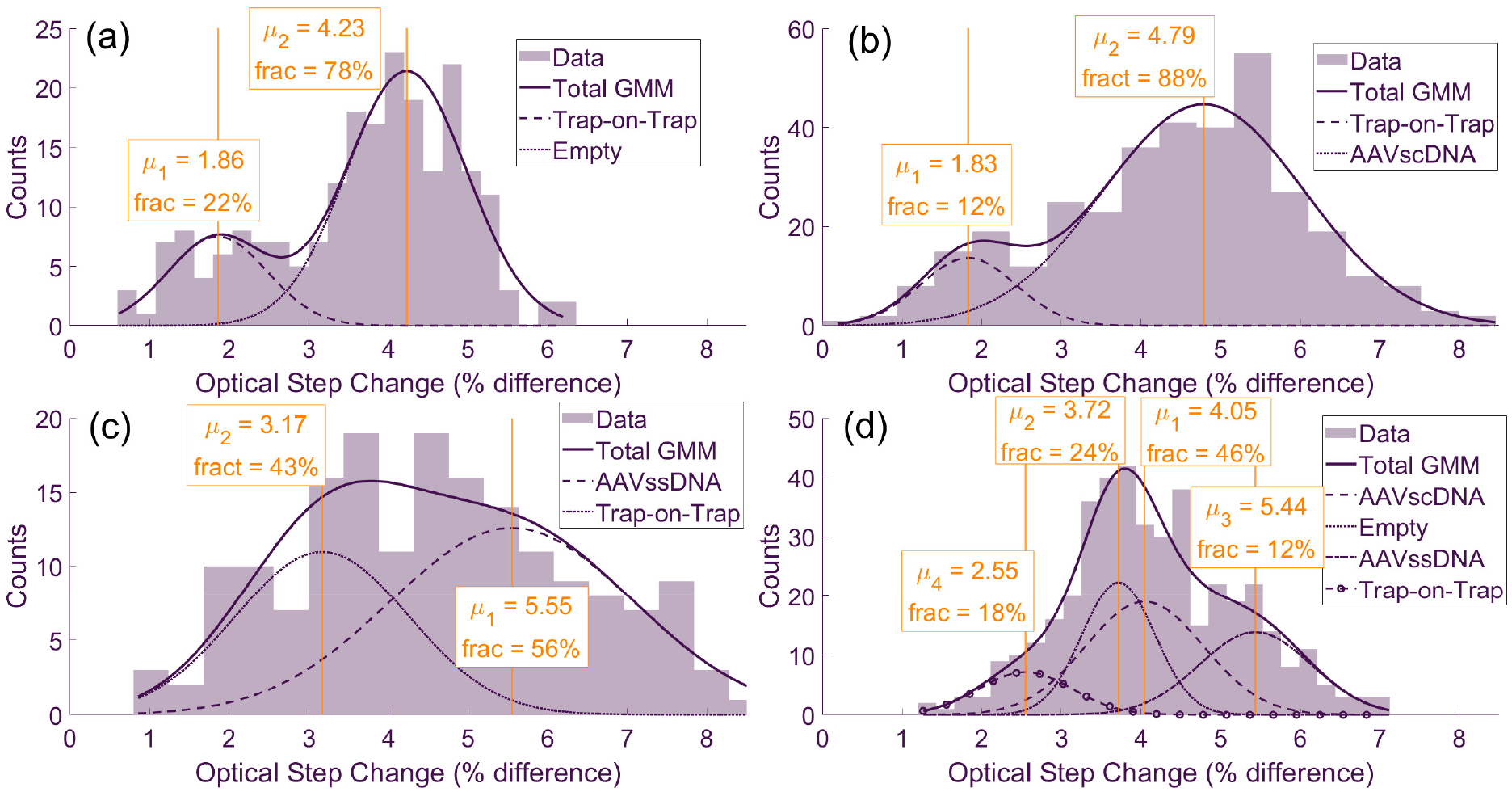
GMM analysis of optical signal step-changes upon optical trapping, compiled into histograms for (**a**) AAV_empty_, (**b**) AAV_ssDNA_, (**c**) AAV_scDNA_ solutions and (**d**) their equimolar mixture.

GMM analysis was applied to the optical step-change histograms for measurements in single analyte species solutions (Figure 3a–c) and an equimolar mixture of AAV_scDNA_, AAV_ssDNA_ and AAV_empty_ (Figure 3d). For the single-species datasets, each distribution contained a dominant Gaussian component at higher step-change values and a smaller component at lower values. The dominant component corresponded to the primary species present in that solution, while the smaller component reflected trap-on-trap events, in which a second particle was captured while another remains trapped. This interpretation for trap-on-trap events was supported by time trace evidence, showing an elevated baseline followed by a new trapping event as a second step. These trap-on-trap events represented local crowding of AAV particles near the sensing region. The single-species optical step size distributions were consistent with expectations from AUC, which served as the ground-truth measurement as previously reported by Thyashan et al. [28]. Specifically, AAV_empty_ (Figure 3a) produced the smallest optical step changes, AAV_scDNA_ (Figure 3b) yielded intermediate values and AAV_ssDNA_ (Figure 3c) generated the largest ones. This trend aligned with previous plasmonic trapping studies using a DNH nanosensor, where particles with greater size typically yielded higher optical step changes [36]. In the case of AAV_ssDNA_ versus AAV_scDNA_, which had near-identical masses, the observed separability on optical step-change signatures between them is likely explained by their difference in DNA conformation inside the AAV particles, which in turn influenced particle deformability and size.

The equimolar mixture (Figure 3d) produced a more complex profile consistent with the presence of the three analyte populations, together with the trap-on-trap component also observed in the single-species datasets. In the GMM analysis for those mixtures, four distinct means were identified, with the smallest component corresponding to trap-on-trap events (Figure 3d). The AAV_scDNA_ and AAV_empty_ populations clustered closely, with the empty capsids producing the most prominent peak shifted to the left, consistent with lower optical step-change values. The AAV_ssDNA_ population appeared as a smaller distribution at higher step-change values. As confirmed by AUC [28], both AAV_ssDNA_ and AAV_scDNA_ contained a fraction of empty capsids, making their equimolar mixture with AAV_empty_ have a higher than one-third of the total population fraction.

### 3.2. Clustering Stability Analysis

**Figure 4.**
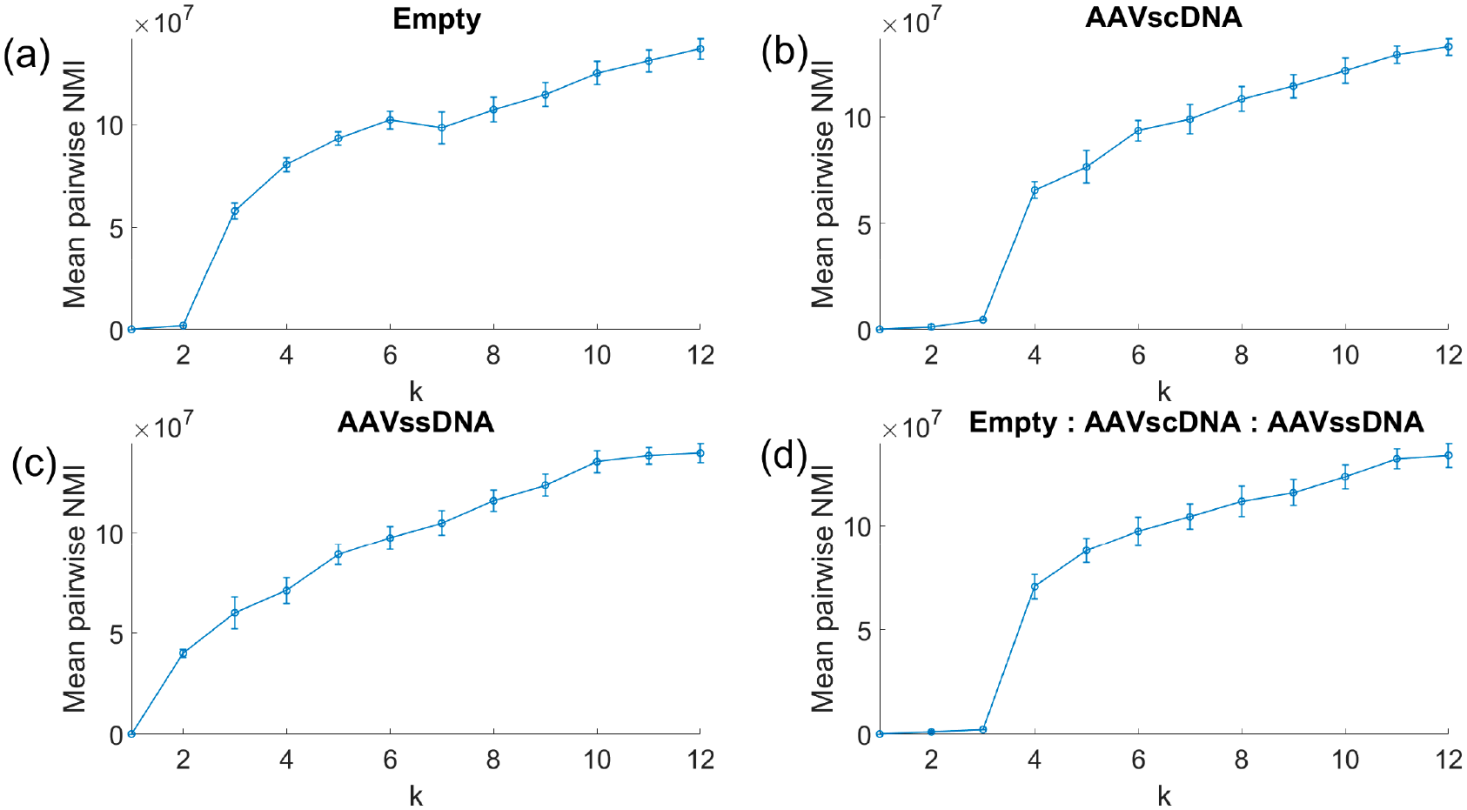
Spectral clustering stability analysis. Inflection points in slope identify the minimum useful cluster numbers *k*: *k* = 3 for AAVempty, *k* = 4 for AAV_ssDNA_, *k* = 2 for AAV_scDNA_, and *k* = 4 for the equimolar mixture.

To further resolve particle subpopulations, spectral clustering was performed across all five data dimensions: optical trap duration, optical step change, reflectance optical step change, electrical entry peak, and electrical exit peak amplitudes. A min-max normalization was applied to all data, and stability was assessed across a range of cluster numbers (Figure 4). Each dataset exhibited a characteristic inflection point in slope at a particular cluster number (Equation (3)), indicating the minimum useful number of clusters. For AAV_empty_ (Figure 4a) the strongest change occurred at *k* = 3 clusters, while the AAV_scDNA_ dataset (Figure 4b) showed a clear inflection at *k* = 4. The AAV_ssDNA_ dataset (Figure 4c) reached its steepest slope at *k* = 2, and the equimolar mixture (Figure 4d) showed a pronounced slope change at *k* = 4.

### 3.3. Data Cluster Maps

Clustering was initially performed across all five data dimensions for the minimum useful cluster number identified by the preceding clustering stability analysis. Clear cluster separation consistently emerged along the two optical step-change dimensions (transmission and reflectance). For the electrical features, the entry and exit spike amplitudes were strongly correlated in amplitude, as expected, with opposite polarity reflecting charge increase and decrease as a particle enters and exits the trap, respectively. Because of their high correlation, only one of the two spike features provided a distinct contribution in cluster separability when compared to other data dimensions. Nevertheless, it was found that electric spikes helped with improved clustering. Trapping duration, by contrast, exhibited more stochastic behavior and was less useful for clustering. Experimentally, trapping durations tended to drift over the course of a run, with longer events occurring as more particles accumulated near the sensing region.

Subsequently, we examined the clusters by starting from the minimum useful number of clusters identified through the stability analysis described above and incrementing *k* gradually. This iterative approach allowed us to track which clusters remained stable as *k* increased and to identify when additional clusters merely subdivided existing ones without improving separability. To distinguish genuine new features from artificial daughter clusters, the trapping current (shown as e–d in Figure 2) was included as an additional feature dimension, providing a physical indicator of distinct particle behavior rather than numerical over-segmentation. Occasionally, small clusters containing only 1–5% of total events were produced. These clusters were generally stable across iterations but appeared as outliers in the data, typically located at the extremes of the feature space or visually isolated from the main populations. Based on this behavior and lack of clear physical correlation, they were retained as stable outliers but excluded from further analyses.

**Figure 5.**
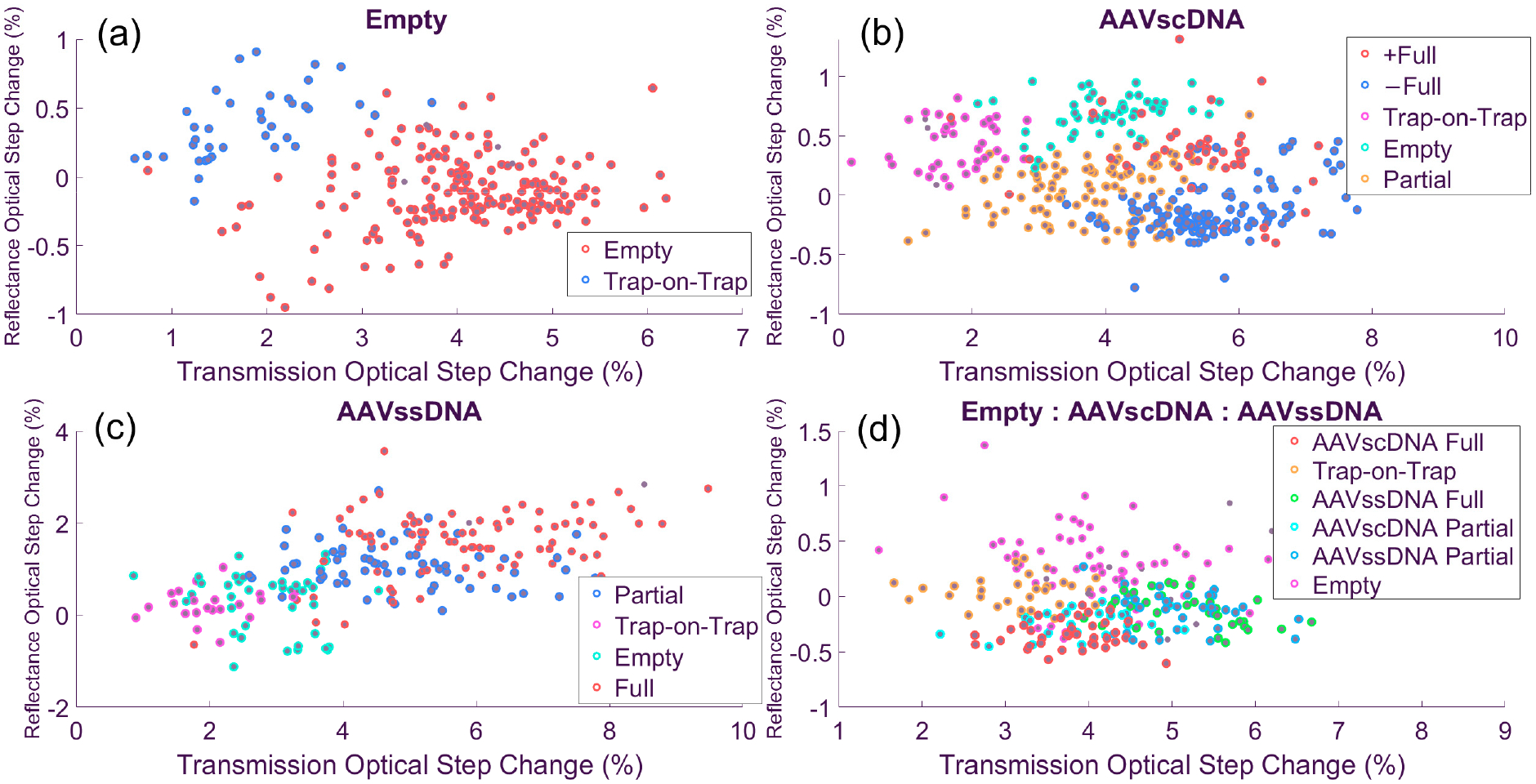
Cluster maps in optical feature space. (**a**) AAV_empty_ separate into aggregate and intact populations. (**b**) AAV_scDNA_ resolve into three clusters, including mirrored positive and negative reference step-change groups. (**c**) AAV_ssDNA_ produce two clusters with similar structure to AAV_scDNA_ but lacking the mirrored subgroup. (**d**) The equimolar mixture resolves into six discernible clusters (with insight from the third, electrical data, dimension) which represent all the cluster types seen in single-analyte solutions.

In Figure 5a, the AAV_empty_ solution separated into two distinct populations: The primary empty capsid cluster and a trap-on-trap cluster. Across 221 recorded events, 183 (82.8%) corresponded to empty capsids (red dots), while 38 (17.2%) represent trap-on-trap events (blue dots). This distribution was consistent with a homogeneous empty capsid solution, where the smaller secondary cluster reflected trap-on-trap interactions rather than additional analyte species. These sensor results were comparable to the AUC results showing 89% of AAV_empty_ particles. It is possible that the trap-on-trap events occurring at the sensor resulted in a small underestimation of the true AAV_empty_ particle population fraction, while a fraction of these events could be due to small amounts of debris in the solution debris or due to aggerates.

In Figure 5b, the AAV_scDNA_ dataset shows a more complex structure. A trap-on-trap cluster appeared at small optical step changes (magenta dots), while a second population aligned with the empty capsid cluster identified in the single-species experiment (turquoise dots). Beyond these two clusters, three additional ones were resolved. Two appeared on the higher end of the optical step-change axis (+Full, −Full; orange and darker blue dots, respectively), both with larger electrical responses (not shown). These two clusters had similar transmission characteristics with their reflection response, which they mirrored. The +Full and −Full clusters likely represented AAV_full_ particles in two different orientations (vertical versus horizontal) inside the optical trap. As the scDNA is expected to be compact and rod-like, this can introduce some asymmetry in the capsid, creating a longer and a shorter symmetry axis [26]. Lastly, a large population at the lower end of the transmission optical step change likely corresponded to AAV_partial_ as they placed in-between the empty and full clusters.

In the AAV_scDNA_ dataset (Figure 5b), excluding trap-on-trap and outlier events, a total of 315 particles were classified into these groups: 44 (14.0%) in the +Full cluster (red dots), 124 (40%) (blue dots), in the −Full cluster, making a total of 54% for AAV_full_. In addition, there were 98 (31%) events for AAV_partial_ (orange), and 49 (16%) for AAV_empty_ (turquoise). When compared with the AUC results summarized in Table 1 [28], which shows 58% for AAV_full_, 32% AAV_partial_, and 10% for AAV_empty_, it is seen that the nanosensor data analysis produced very comparable population fractions. One possible explanation for this discrepancy seen for the AAV_empty_ fraction is that these particles tend to be ‘stickier,’ as observed in the time traces, and are more likely to participate in trap-on-trap events that were then excluded from the nanosensor analyses. Supporting this interpretation, comparisons of the time traces showed that empty capsid events had more poorly defined optical trapping shape, generally with a shallower slope (not shown), than those of full or partial capsids, suggesting that their trapping dynamics may have biased the measured distribution toward a higher fraction of empties.

In Figure 5c, the AAV_ssDNA_ dataset separates into four clusters: AAV_full_ (red dots), AAV_partial_, (blue dots), trap-on-trap (magenta), and AAV_empty_ (turquoise), with a total of 163 events. Of these, 60 (37%) corresponded to AAV_full_, 65 (40%) AAV_partial_, and 38 (23%) to AAV_empty_, with the remaining events classified as trap-on-trap. When compared with AUC results (Table 2) [28], where the expected distribution was 52% for full capsids, 33% for partials, and 14% for empties, the nanosensor results show a modest overrepresentation of empties and a corresponding underrepresentation of full capsids. This shift likely reflected the tendency of AAV_empty_ to participate more frequently in trap-on-trap and aggregation events due to their increase capsid ‘stickiness.’ The latter is also evidenced in Thyashan et al. [28], where the empty capsids were the only particle type that showed a small but detectable (~6%) aggregate population fraction.

In Figure 5d, the equimolar mixture solution revealed a more complicated structure due to the greater number of AAV types present and their possible interaction permutations. Several distinct clusters were resolved, including AAV_empty_, a trap-on-trap cluster, and four populations with different DNA load status corresponding to AAV_scDNA_ and AAV_ssDNA_ (fully and partially loaded for each category). These assignments were made primarily using optical step-change features, with electrical signals providing secondary confirmation to distinguish between overlapping groups. A total of 184 particles were analyzed: 57 events fell into the AAV_empty_ cluster (31.0%, magenta dots), while the AAV_scDNA_ partial and full clusters contained 23 (12.5%, turquoise dots) and 36 (19.6%, red dots). Also, the AAV_ssDNA_ partial and full clusters contained 35 (19.0%, blue dots) and 33 (17.9%, green dots), respectively.

When compared with expected values derived by proportionally combining the AUC results of the single-species datasets (41% AAV_empty_, 11% scDNA AAV_partial_, 19% scDNA AAV_full_, 12% ssDNA AAV_partial_, and 17% ssDNA AAV_full_) [28], the nanosensor results showed a slight underrepresentation of the AAV_empty_ (31% measured vs. 41% expected) and a corresponding modest increase in the partial and full populations. The apparent reduction in empties likely reflected the exclusion of the trap-on-trap cluster from this analysis, as both debris and empty particles were more likely to contribute to trap-on-trap events. Overall, however, the nanosensor data reproduced the expected balance of empty, partial, and full capsid loads in the equimolar preparation, with only modest deviations attributable to trapping dynamics. These results are summarized in Table 3.

### 3.4. Special Cases in Optical and Electrical Time-Series Data Features

**Figure 6.**
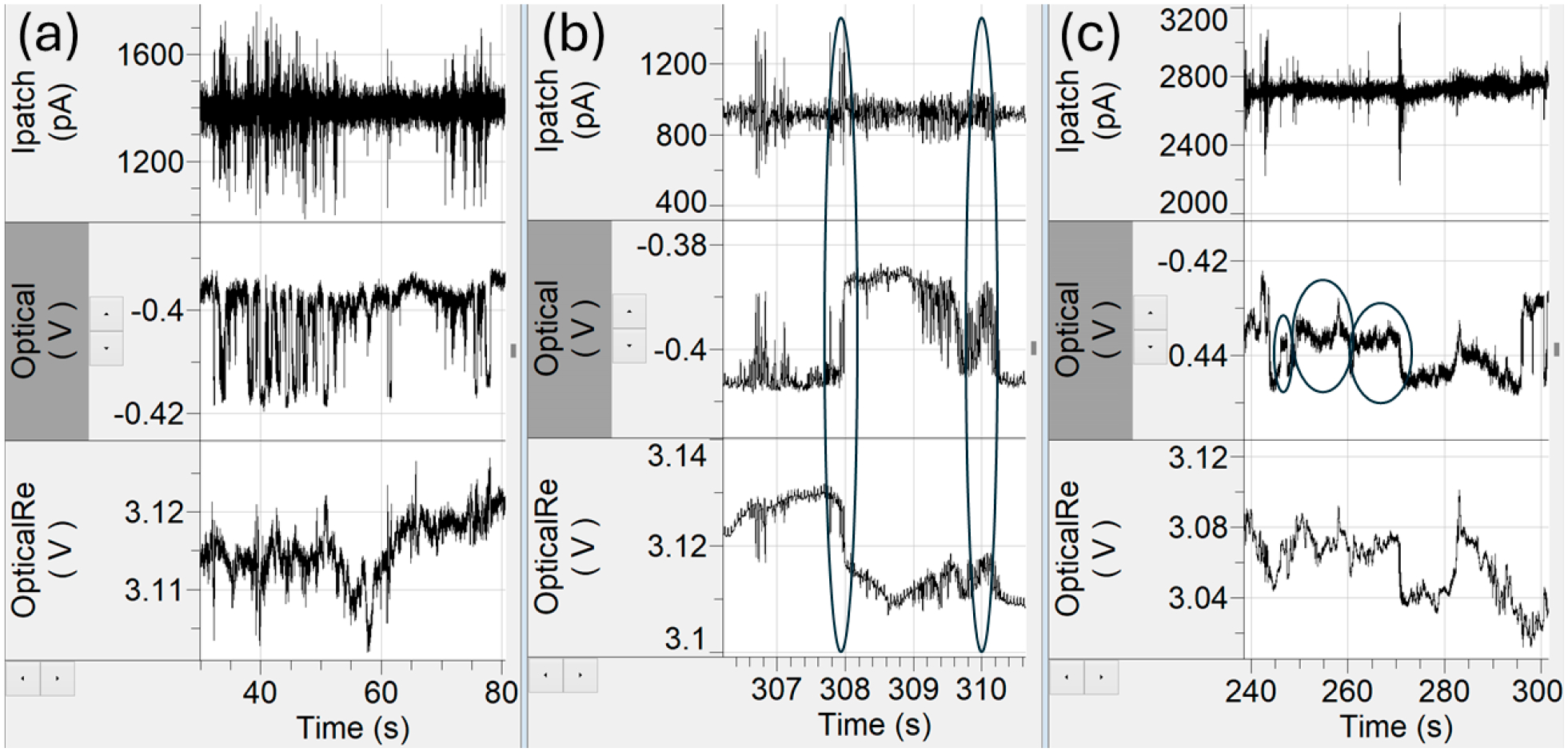
Special cases in event analysis. (**a**) Rapid entry–reentry events create the risk of potentially overcounting trapping events. (**b**) A gradual exit, characterized by less steep ramps and more noise (ovals), obscure electrical spikes and distorted event magnitudes. (**c**) Debris events (ovals) with untypically small optical step changes between 0.1% and 1.5%.

Several special cases were noted during event analysis, which are important to describe for the correct interpretation of the time series data collected by the nanosensor:

Rapid entry–reentry events (Figure 6a) were characterized by short duration step-change patterns in optical intensity, interpreted as AAV particles moving repeatedly in and out of the optical trap. Each reentry produced a corresponding electrical spike upon entry and exit. At first glance, these could be classified as multiple trapping events (“string-of-pearls” behavior) but based on prior observations in similar plasmonic trapping systems [22], these behaviors most likely reflected a single trapped particle intermittently entering and leaving the high light intensity region rather than sequential trapping of multiple particles. Generally, traps occur in small groups with periods of no trapping. In the rapid entry–reentry events, they instead appear as a lot of short duration traps back-to-back with optical trap step sizes and electrical spike amplitudes being very similar instead of showing variability as one would expect if these were independent particles. Without identifying these events as a single particle in a rapid entry–reentry event, they can be counted as upwards of 30 events which can potentially skew the data toward that particle. Time gate filters that look at temporal separation between traps can be used to identify and remove these events. The rapid entry–reentry events can account for upwards of 20% of the total trapping data. However, we observed the AAV_empty_ and equimolar experiments had a higher occurrence of the rapid entry–reentry events than the AAV_scDNA_ and AAV_ssDNA_ solutions.

Gradual entrance and exit in or out of the optical trap (Figure 6b) were also observed. Normally, particles entered the trap with a sharp, step-like, increase in optical intensity and likewise upon exit, each event was accompanied by an electrical spike. In contrast, gradual entry/exit events exhibited a gradual ramp-up or ramp-down in optical intensity, often accompanied by higher-frequency fluctuations resembling miniature entry–reentry structures. These events accounted for approximately 2–10% of the total trapping events in each dataset. While reliable values for event duration and optical step change could still be extracted, the asymmetric electrical responses (e.g., an exit spike not being as sharp as the entrance spike) complicated interpretation. The reduction in electrical spike amplitude is largely due to the ion displacement being spread out over a longer period, as particle translocation was slower in those cases.

Finally, small debris events were identified (Figure 6c), which were characterized by unusually small step changes in optical transmission intensity, typically between 0.1% and 1.5%, far below the levels observed for capsid populations in the 3–7% range. Such events likely arose from residual proteins, degraded capsid fragments, or stray nucleic acids not encapsulated within AAV capsids [37].

## 4. Discussion

Traditional plasmonic trapping approaches rely almost exclusively on a detected optical step change as a proxy for particle size [38,39]. Because the optical step change amplitude is dependent on multiple physical properties of a particle (size, shape, and refractive index—a proxy for density), much of the underlying AAV physical property heterogeneity is effectively lost. As a result, populations that differ meaningfully in structure may collapse onto overlapping measurement values, masking critical distinctions. This work highlights both the strengths and limitations of the plasmonic nanopore approach as a novel way of probing additional data dimensions for characterizing AAV DNA load status and type. As expected, the single-species analytes generally produced Gaussian-like distributions, but each also contained secondary distribution components (Figure 3). Closer inspection of the time-series data suggested that one of these component Gaussians in each dataset represented complex trapping events, referred to as trap-on-trap events (Figure 3). Such events were consistently observed across the empty, AAV_scDNA_, and AAV_ssDNA_ datasets, as well as in the equimolar mixture and they are a result of the experimental conditions used in this work.

The relative positions of the dominant population fraction Gaussians in Figure 3 support the expected size hierarchy of capsid populations. Empty capsids yielded the smallest mean optical step change (4.23%), AAV_scDNA_ particles were intermediate (4.79%), and AAV_ssDNA_ particles produced the largest values (5.55%). This ordering is consistent with the physical characteristics of the genomes: The AAV_ssDNA_ construct is single-stranded and therefore less compact. On the other hand, the self-complementary AAV_scDNA_ construct, could adopt denser packing. Empty capsids, lacking DNA load, appeared at the smallest size values since they had no negatively charged DNA load pushing the capsid radially outwards. In addition, the lack of DNA load reduced the charge density of the capsid [40,41]. Without the negatively charged DNA, empty AAV9 capsids have a higher isoelectric point (pI), around 6.3, whereas for full capsids it is 5.9. At physiological pH (7.4), where experiments were performed, the difference between the pI and the solution pH was therefore smaller for empty relative to full capsids, resulting in lower net negative charge, and a greater tendency to self-interact and aggregate [42]. Being closer to the pI point was consistent with lower solubility and the more ‘sticky’ behavior observed at the nanosensor. In the equimolar mixture (Figure 3d) four Gaussian components were observed, corresponding to the three reference populations plus the trap-on-trap events. However, the overlap between AAV_scDNA_ and AAV_ssDNA_ distributions underscored the limitation of optical-only sensing when particles have similar mass.

Where our sensor demonstrated its true advantage was in its ability to integrate multiple data dimensions to improve AAV species separability. By combining optical and electrical signals with a reference channel that captured backscattered light, the platform moved beyond one-dimensional descriptors and revealed asymmetries in particle scattering. This effect was particularly apparent in the AAV_scDNA_ dataset (Figure 5b), where clustering revealed mirror-image populations that were tagged as +Full and −Full AAV_scDNA_. The emergence of these paired populations suggested an orientation-dependent light scattering response: The self-complementary genome likely imposed greater internal rigidity, producing a slightly flattened, or oblate, capsid geometry. The broader and narrower faces of this geometry could interact differently with the plasmonic field, leading to measurable differences in backscattered light. Because optical paths to and from the trap are not symmetric, some scattered light is redirected off-axis and lost, resulting in unequal total intensity between orientations. The difference in population sizes between +Full and −Full clusters therefore reflected a preferred or more stable scattering configuration rather than a defined geometric orientation. The absence of this asymmetry in the AAV_partial_ or AAV_empty_ species suggests that it is a unique feature of the fully loaded self-complementary capsid and its associated scDNA rigidity.

Surface charge also influenced how deeply a capsid would settle inside the optical trap: Because the Au layer was not grounded, or held at a defined potential, mirror charges were induced by the applied electric fields, which could repel the more highly charged particles. Furthermore, electrostatic particle repulsion could have been influenced by the trapping laser itself, as the local E-field at the DNH Au cusps could establish a locally focused plasmon of higher electron density contributing to the mirror-charge response of the sensor. However, laser fluctuations and external vibrations, potentially introduced additional drift and noise into the measurements.

Another factor contributing to the observed asymmetries was the geometry of the AAV9 capsid itself, which is a function of its deformability [43]. Depending on how an individual capsid orients within the trap—oblate versus prolate cross-sections—the optical signatures may shift [44]. These shape differences alter the light-scattering profiles that are used to deduce the apparent particle volume. We hypothesize that the +Full and −Full populations seen in Figure 5b were driven by the relative structural rigidity of the scDNA capsid load, whereas no such distinction was made in Figure 5c for AAV_ssDNA_ because its capsid was less asymmetric.

The equimolar mixture was intended as a validation test, designed to demonstrate whether the nanosensor could resolve an expected distribution of roughly one-third AAV_ssDNA_, one-third AAV_scDNA_, and one-third empty capsid population fractions. A slightly higher weighting toward the empty species was expected, because both the AAV_ssDNA_ and AAV_scDNA_ solutions would inevitably also contain an empty capsid fraction. However, comparison with AUC data revealed that the equimolar solution data obtained from the nanosensor was far more complex than anticipated, producing a feature-rich dataset that extended beyond the simple tripartite expectation. In practice, a larger number of measured events would be required to improve on statistical uncertainty of population fractions and fully characterize this expanded feature set. The most important outcome from the equimolar experiments was that our sensor could indeed differentiate between populations in a complex mixture, but at the cost of increased ambiguity at interpreting the identity of higher numbers of clusters and a higher incidence of trap-on-trap events, possibly due to the competition between different AAV types to enter the trap. These observations highlight the dual nature of the platform: enhanced dimensionality enables richer resolution but also demands more rigorous controls.

Lastly, a few special cases in the time-series data were noted, which highlighted additional factors that must be considered when interpreting plasmonic nanopore data. Events with very small optical step changes likely represented free proteins, partial capsids, or free-floating DNA. It is possible that this debris transiently adhered to the nanosensor non-specifically [45], or trapped and un-trapped quickly due to its smaller mass range. In addition, rapid on-off step-changes in optical signals, interpreted as rapid entry–reentry events, may have reflected unstable confinement near the nanopore sensor. Alternatively, as our nanosensor is known to create a traffic jam of analytes around the optical trap [22], it is possible that multiple particles could line up like a ‘string of pearls’ and sequentially pass through the nanopore. The multi-dimensional feature space makes it easier to distinguish between the former and the latter cases making a ‘string of pearls’ scenario unlikely. Gradual particle entry or exit events from the optical trap likely corresponded to capsids interacting with the nanopore during translocation. It is also possible that particles moved laterally between the two optical hot spots of the DNH cusps [38]. These positional effects could modulate transmission and backscatter amplitudes, adding variability to weak exit events. The gradual traps were usually not tagged as events due to poor definition of the leading edge of the trap and low electrical response. Weak exit traps were included as we could ensure there was a clearly defined optical step change and trapping current.

Lastly, drift in optical alignment across long experiments and noise introduced by electrical alignment contribute to baseline variability between runs. Although electrical channel data do improve cluster separability, they are not yet reliable as a standalone discriminator due to higher noise. Future methodological refinements, such as introducing controlled flow to fight trap-on-trap analyte settling, improving optical trap stability with automated alignment, coating the Au layer with a hydrophilic coating [46], using carefully designed Faraday cages [47], and implementing new protocols for more accurate baseline corrections in optical and electrical data, will help mitigate sources of uncertainty in mixed-population measurements.

## 5. Conclusions

This work demonstrated that the use of a plasmonic nanopore sensor shows significant promise as a higher-sensitivity QC platform for AAV9 gene delivery vectors. Existing methods provide limited discriminatory capacity when attempting to resolve closely related capsid species, such as AAV_full_ and AAV_partial_, particularly when their masses overlap. With further refinements—such as removal of aggregates from the analysis pipeline and expansion of sample sizes—we anticipate even clearer differentiation between these species. In more realistic scenarios, where partial loads are likely to be appreciably smaller in mass than fully packaged capsids, the separation should be even more pronounced. Comparisons between single-analyte samples and the equimolar mixture revealed a distribution that closely mirrored the expected ratios, validating this approach for use in more complex mixtures. Several technical limitations remain, which were discussed. Looking forward, this approach can be expanded to incorporate alternating current interrogation [48], which may differentially probe between the inside and outside of a nanoparticle [49] (DNA load versus the capsid and its surroundings) to further improve separation between AAV species. Such developments would push this nanosensor system closer to an all-in-one impedance-like spectroscopy for nanoparticle quality control, enabling precise, low-volume characterization of viral vectors in both research and manufacturing settings.

While single-particle resolution is a core strength of this platform, its current throughput is modest, typically 100–300 events per hour under the trapping conditions used in this study. Nevertheless, several clear paths exist for scaling throughput to levels more suitable for QC workflows. These include adjusting laser power and command voltage to shorten trapping durations while retaining robust optical–electrical signatures and parallelizing the architecture through arrays of plasmonic nanopores operating simultaneously. Although these engineering developments lie beyond the scope of the present manuscript, they represent a practical roadmap toward higher-throughput implementations compatible with industrial needs.

The optical sensing component of this technology is being considered for potential future applications in protein drug discovery, protein dynamics, and interaction analysis [50], and has even been tested for egg white protein composition [51]. The potential of the combined optical electrical nanosensor technology presented in this manuscript has an even broader potential range of possible applications due to the increased discrimination capacity offered by combining information from concurrently acquired multi-modal data. Although this technology has not yet been explored more broadly, it could in principle find use cases to any therapeutic nanoparticle and more broadly to the detection of contaminants such as microplastics among many possible applications.

## Figures and Tables

**Table 1.** Observed cluster distributions for single-analyte and AAV_scDNA_ experiments comparing AUC-determined population fractions to the ones determined by clustering the nanosensor data.

AAV_scDNA_ Population Fractions	Empty	Partial
Expected (AUC)	10%	32%
Measured (Nanosensor)	16%	31%

**Table 2.** Observed cluster distributions for single-analyte and AAV_ssDNA_ experiments comparing AUC-determined populations fractions to the ones determined by clustering the nanosensor data.

AAV_ssDNA_ Population Fractions	Empty	Partial	Full
Expected (AUC)	14%	33%	52%
Measured (Nanosensor)	21%	35%	44%

**Table 3.** Observed cluster distributions for an equimolar mixture of AAV_empty_, AAV_scDNA_, and AAV_ssDNA_ experiments comparing AUC-determined population fractions based on single-analyte solution measurements to the ones determined by clustering the nanosensor data from the complex mixture.

Equimolar Mixture Population Fractions	Empty	AAV_scDNA_ Partial	AAV_scDNA_ Full	AAV_ssDNA_ Partial	AAV_ssDNA_ Full
Expected (Single-Analyte AUC)	41%	11%	19%	12%	17%
Measured (Nanosensor)	31%	13%	20%	18%	18%

## Data Availability

The raw data supporting the conclusions of this article will be made available by the authors on request.
